# Anti-Remodeling Effects of Rapamycin in Experimental Heart Failure: Dose Response and Interaction with Angiotensin Receptor Blockade

**DOI:** 10.1371/journal.pone.0081325

**Published:** 2013-12-03

**Authors:** Kalkidan Bishu, Ozgur Ogut, Sudhir Kushwaha, Selma F. Mohammed, Tomohito Ohtani, Xiaolei Xu, Frank V. Brozovich, Margaret M. Redfield

**Affiliations:** Division of Cardiovascular Diseases, Mayo Clinic, Rochester, Minnesota, United States of America; Albert Einstein College of Medicine, United States of America

## Abstract

While neurohumoral antagonists improve outcomes in heart failure (HF), cardiac remodeling and dysfunction progress and outcomes remain poor. Therapies superior or additive to standard HF therapy are needed. Pharmacologic mTOR inhibition by rapamycin attenuated adverse cardiac remodeling and dysfunction in experimental heart failure (HF). However, these studies used rapamycin doses that produced blood drug levels targeted for primary immunosuppression in human transplantation and therefore the immunosuppressive effects may limit clinical translation. Further, the relative or incremental effect of rapamycin combined with standard HF therapies targeting upstream regulators of cardiac remodeling (neurohumoral antagonists) has not been defined. Our objectives were to determine if anti-remodeling effects of rapamycin were preserved at lower doses and whether rapamycin effects were similar or additive to a standard HF therapy (angiotensin receptor blocker (losartan)). Experimental murine HF was produced by transverse aortic constriction (TAC). At three weeks post-TAC, male mice with established HF were treated with placebo, rapamycin at a dose producing immunosuppressive drug levels (target dose), low dose (50% target dose) rapamycin, losartan or rapamycin + losartan for six weeks. Cardiac structure and function (echocardiography, catheterization, pathology, hypertrophic and fibrotic gene expression profiles) were assessed. Downstream mTOR signaling pathways regulating protein synthesis (S6K1 and S6) and autophagy (LC3B-II) were characterized. TAC-HF mice displayed eccentric hypertrophy, systolic dysfunction and pulmonary congestion. These perturbations were attenuated to a similar degree by oral rapamycin doses achieving target (13.3±2.1 ng/dL) or low (6.7±2.5 ng/dL) blood levels. Rapamycin treatment decreased mTOR mediated regulators of protein synthesis and increased mTOR mediated regulators of autophagy. Losartan monotherapy did not attenuate remodeling, whereas Losartan added to rapamycin provided no incremental benefit over rapamycin alone. These data lend support to investigation of low dose rapamycin as a novel therapy in human HF.

## Introduction

Over six million Americans have heart failure (HF) and while treatment with renin-angiotensin-aldosterone system (RAAS) antagonists and β-adrenergic antagonists improve outcomes in HF, progressive cardiac remodeling and dysfunction occur on standard therapy and outcomes are poor [1]–[3]. Cardiac transplantation, left ventricular assist devices and in some patients, correction of valvular abnormalities are the only life-extending treatments for advanced HF [1]–[3]. However, the majority of HF patients are not candidates for these invasive procedures. The need for novel HF therapies has spawned interest in cell and gene therapies for HF [4], [5], but these strategies are still highly investigational. Small molecules targeting other pathways involved in pathophysiologic remodeling remain attractive candidates for novel HF therapies.

Mechanistic target of rapamycin (mTOR) is a kinase that plays a significant role in broad signaling networks related to protein synthesis, cell cycle progression, autophagy and actin organization (reviewed in [6]). mTOR may associate into two distinct signaling complexes, mTORC1 and mTORC2. AKT, via inhibition of tuberous sclerosis complex, is a key regulator of mTORC1 activity in both physiologic and pathophysiologic hypertrophy and evidence suggests that the time course of AKT activation (transient vs sustained) may be a key differentiator of adaptive versus maladaptive remodeling [7], [8]. mTORC2 may also regulate growth via activation of AKT and thus, mTORC1. mTORC1 regulates protein synthesis via a number downstream effectors. Among these, mTORC1 phosphorylation of S6 kinase 1 (S6K1) regulates different effectors mediating Cap-dependent translocation, translation elongation, mRNA biogenesis and, via phosphorylation of ribosomal protein S6 (S6), ribosome biogenesis. In concert with its role in protein synthesis, mTORC1 localization and signaling is also involved in the regulation of autophagy [9]. mTORC1 signaling is sensitive to pharmacological inhibition by rapamycin, a macrolide that binds FK-binding protein 12 (FKBP12) to form a drug-protein complex that can bind to and inhibit mTOR present within mTORC1. While FKBP12-rapamycin does not bind mTOR present within an mTORC2 complex, there is evidence that prolonged rapamycin therapy may also inhibit mTORC2 by limiting integration of *de novo* synthesized mTOR into mTORC2. This occurs in a cell specific manner *in vitro* and in normal heart tissue after rapamycin administration *in vivo* [10], although the dose used for *in vivo* studies (10 mg/kg intraperitoneally, IP) was quite high relative to other *in vivo* studies (2 mg/kg IP, below).

Rapamycin also inhibits cytokine stimulated lymphocyte proliferation and is a potent immunosuppressive agent widely used in transplantation. In this setting, its therapeutic and adverse effects are dose related and thus, blood rapamycin levels are used to guide dosing [11]. However, rapamycin has been shown to ameliorate humorally mediated myocyte hypertrophy *in vitro* [12]–[14] and progressive cardiac remodeling and dysfunction in response to mechanical stress or cardiac injury *in vivo* [12], [15]–[19]. These studies suggest that rapamycin treatment may represent a novel therapeutic strategy in HF.

The potential to preserve favorable cardiac effects of rapamycin while limiting immunosuppressive effects by minimizing dose has not been explored. Further, as the hypertrophic response to mechanical stress, cardiac injury and neuroendocrine activation in HF [20] involve up-stream regulators such as the angiotensin II receptor, the relative and incremental effect of an angiotensin receptor blocker (ARB) to rapamycin must be defined prior to consideration for clinical translation in HF.

Accordingly, the primary objective of this study was to determine if rapamycin treatment in murine experimental HF produced by transverse aortic constriction (TAC) results in amelioration of cardiac remodeling and dysfunction at blood rapamycin levels at (target dose) or below (low dose) those used for primary immunosuppression. Additionally, we sought to determine the relative and incremental effects of ARB to rapamycin in HF. To gain insight into the time course of mTOR and angiotensin activation in the progression of HF, we defined the effect of rapamycin, ARB and their combination on TAC mice with established remodeling but without HF (compensated hypertrophy, TAC-COMP). Pathology, functional studies and gene expression profiles were used to characterize the effect of rapamycin and ARB on cardiac structure and function. Abundance of total and site specific phosphorylated S6K1 and S6 were used to assess the effect of HF and rapamycin treatment on mTOR regulated protein synthesis whereas the abundance of LC3B-II was used to assess autophagy. Studies in normal mice were performed to document the rapamycin administration route and dose relationship to blood rapamycin levels and to verify effects of rapamycin treatment on mTOR regulated effectors of protein synthesis and autophagy in the absence of HF.

## Materials and Methods

### Ethics statement

All work was performed in accordance with the American Physiological Society principles for ethical treatment of animals, using a protocol approved by the Mayo Clinic Institutional Animal Care and Use Committee.

### Rapamycin dosing studies

For intraperitoneal (IP) administration, pure rapamycin powder (provided by Wyeth, Madison, NJ) was administered to eight weeks old male C57BL/6 mice (Jackson Laboratory, Bar Harbor, ME) for two weeks at a dose of 2 mg/kg/day [21]. For oral (PO) administration, Rapamune® tablets (Pfizer, New York, NY) were crushed and mixed in ∼0.25 teaspoons of Nutrical feed supplement (Fort Worth, TX) each morning in order to achieve 2, 5 or 8 mg rapamycin/kg/day dosing. Drug consumption was validated daily. After two weeks of treatment, trough rapamycin levels were measured by High-Performance Liquid Chromatography/Tandem Mass Spectrometry (HPLC-MS/MS) in the Mayo Clinic Core Laboratory.

### Rapamycin treatment in normal mice

To assess rapamycin effects on cardiac structure and downstream signaling independent of the HF state, normal mice were treated with target dose rapamycin for 2 weeks and tissue harvested for pathology and protein studies. To confirm autophagy in rapamycin treated normal mice, a subset of rapamycin treated mice received 10 mg/kg chloroquine IP (Sigma Aldrich, St. Louis, MO) administered 4 hours prior to sacrifice [22]. The lysosomotrope chloroquine prevents acidification of the lysosome and lysosomal protein degradation, prompting accumulation of the autophagy marker LC3B-II under pro-autophagic conditions [23]. Therefore, under the pro-autophagic conditions induced by rapamycin, LC3B-II accumulation is expected and would be additionally increased by concomitant chloroquine treatment.

### Rapamycin treatment in murine experimental HF and compensated hypertrophy

Eight weeks old male C57BL/6 mice were subjected to TAC as previously described [24], [25]. SHAM mice underwent an identical procedure without placement of a suture to produce TAC. As previously described, TAC produces a variable phenotype [26] with some mice developing a HF phenotype characterized by severe systolic dysfunction and pulmonary congestion whereas others develop a compensated phenotype with hypertrophy but without severe systolic dysfunction or pulmonary congestion. We have previously demonstrated that reduced ejection fraction (EF<65%) at three weeks post-TAC reliably predicts the ultimate HF phenotype [26]. Thus, at 3 weeks post-TAC, mice underwent echocardiography and those with established remodeling (defined as systolic dysfunction EF<65%) entered the HF arm of the study (HF) while mice with preserved EF entered the compensated hypertrophy (TAC-COMP) arm of the study.

Oral treatment regimens were based on data from rapamycin dosing studies as above and doses of losartan used in previous murine HF studies [27]. Treatment groups included placebo (Nutrical alone), 8 mg/kg/day rapamycin (target dose), 4 mg/kg/day rapamycin (low dose), 30 mg/kg/day losartan (Sigma Aldrich, St. Louis, MO), or 8 mg/kg/day rapamycin plus 30 mg/kg/day losartan. For TAC-COMP mice, treatment regimens were identical to those in TAC mice with HF. Treatment duration was designed to assess chronic effects and was continued for 6 weeks in all study groups. Overall study duration was 9 weeks, which consisted of a 3 week post-TAC / pre-treatment interval and subsequent 6 weeks of therapy.

### Echocardiography, hemodynamic analyses and tissue harvest

Mice underwent 2-dimensional echocardiography (GE Healthcare, Milwaukee, WI) with a 13-MHz probe under light isoflurane anesthesia (0.5% to 1.0% v/v) administered via a nose cone. All measurements were made by an operator blinded to study group. The gradient across the TAC constriction was assessed by pulsed-wave Doppler and reported as aortic flow velocity which is proportional to the severity of stenosis and level of systolic function [25]. At study end, immediately after echocardiography, isoflurane-anesthetized mice were intubated and mechanically ventilated (Hugo Sachs Elektronik, Hugstetten, Germany). A manometer tipped catheter (Millar Instruments, Houston, TX) was inserted into the LV via the right carotid artery to measure LV pressure [25]. For tissue harvest following *in vivo* measurements, ventilation was ceased and mice were sacrificed by administration of high dose (5%) isoflurane anesthesia, consistent with the guidelines of the Mayo Clinic Institutional Animal Care and Use Committee. Dissected organs were quickly weighed prior to flash-freezing with liquid nitrogen for RNA transcript and protein analyses, or formalin fixed and paraffin embedded for histomorphometry.

### Quantitative real-time reverse-transcription polymerase chain reaction

Total RNA was extracted and reverse transcribed to complementary DNA using the iScript complementary DNA synthesis kit (Bio-Rad Laboratories, Hercules, CA). Complementary DNA was amplified and levels of gene expression were quantified by real-time quantitative polymerase chain reaction (TaqMan Gene Expression Assays and Universal Probe library Gene Assays). Primers for transcripts reflecting hypertrophy (atrial natriuretic peptide) and fibrosis (collagen type I and III) were used (Roche Applied Science, Indianapolis, IN).

### Histomorphometry

Interstitial fibrosis was assessed on picrosirius red stained full short-axis LV sections using a semiquantitative visual analog fibrosis score (0 = no fibrosis, 1 = trivial fibrosis, 2 = mild fibrosis, 3 = moderate fibrosis, and 4 = severe fibrosis) for each of the four LV quadrants yielding a total score between 0 and 16. Standard examples from previous studies showing each grade of fibrosis were used to increase consistency of scoring. Given the variable perivascular fibrosis (within an LV section) and the variable number of vessels in any given section, analyses were focused on interstitial fibrosis remote from vessels. When the quality of staining in parts of a slide was suboptimal, the remaining quadrants were averaged. Cardiomyocyte cross-sectional area was measured on hematoxylin/eosin LV sections [28]using ImageJ software (ImageJ, NIH, Bethesda, MD). Up to 50 cells in cross-section per slide were selected for area analysis. Both fibrosis and cardiomyocyte cross-sectional area assessments were performed blinded to study group.

### Western blotting

Proteins were extracted by homogenization of tissues on ice using a buffer comprised of 50% glycerol, 0.4 M Bis-Tris pH 7, 4% LDS, 2 mM EDTA, 0.075% Coomassie G250 and 0.25% Phenol Red. Prior to sample loading, DTT was added to 50 mM. Protein extracts were resolved by Bis-Tris SDS-PAGE and transferred to Hybond PVDF membrane (GE Healthcare, Piscataway, NJ). Antibodies used identified S6K1, pThr389 S6K1, S6, pSer235/236 S6, microtubule-associated protein light chain 3B (LC3B-I and II) (Cell Signaling Technology, Danvers, MA). Cardiac sarcomeric α-actin was used to standardize protein loading across different lanes (Sigma Aldrich, St. Louis, MO). Secondary antibodies conjugated to Cy3 or Cy5 dyes were used to visualize the total protein and phosphorylation signals, and were quantified by densitometry using a Typhoon 9410 scanner and accompanying ImageQuant TL software. All samples were normalized to a standard LV homogenate sample for reliable comparison across gels.

### Statistical analyses

Results are expressed as mean ± SEM. Statistical analyses were performed with the SPSS statistical software package (Version 19.0, SPSS Inc, Chicago, IL). Comparison across groups was performed using 1-way analysis of variance with post hoc comparisons by Dunnett's post hoc test and unpaired Student's t test where appropriate without adjustment for multiple comparisons. A p value of <0.05 was considered significant.

## Results

### Rapamycin administration route, dose and blood levels

**Figure 1:**
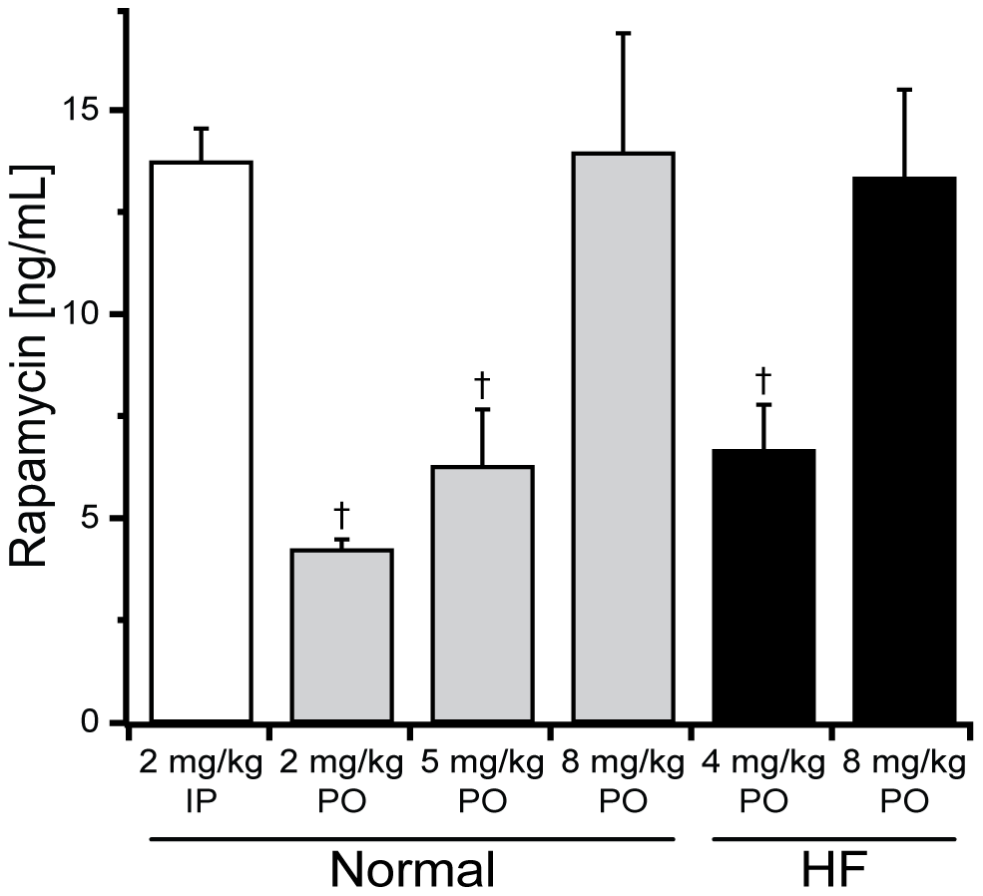
Serum rapamycin levels in response to dosing and administration routes. Mice were dosed with 2, 4, 5 or 8/kg/day rapamycin through intraperitoneal (IP) or oral (PO) administration routes. Serum levels from 2 (n = 5), 5 (n = 6) and 8 (n = 8) mg/kg/day PO administration were dose dependent, with the latter approaching serum levels achieved with 2 mg/kg/day IP administration (n = 5). Serum rapamycin levels for HF mice given 8 mg/kg/day PO (n = 13) dosing were not different than that for normal mice, whereas 4 mg/kg/day PO (n = 6) showed reduced serum levels as expected. Data are mean ± SEM. †: *P*<0.05 vs. 2 mg/kg/day IP.

Rapamycin administered at 2 mg/kg IP has been shown to ameliorate remodeling in a murine model [21]. This dose achieved trough serum rapamycin levels (13.8±0.8 ng/mL) similar to those targeted when rapamycin is used for primary immunosuppression in human transplant recipients (7–15 ng/ml; [11]). Similar levels were achieved with PO administration at 8 mg/kg/day (14.0±2.9 ng/mL, Fig 1), reflecting differences in bioavailability with IP and PO administration. Both the 2 and 5 mg/kg/day PO doses resulted in serum levels below those targeted for immunosuppression and significantly below that achieved from 8 mg/kg/day PO dosing (Fig 1). Based on these studies in normal mice, doses of 4 (low dose) and 8 (target dose) mg/kg/day were chosen for HF and TAC-COMP studies. The trough serum concentrations achieved with similar rapamycin doses were not different in HF versus normal mice (Fig. 1).

### Effects of rapamycin in normal mice

Following 2 weeks of treatment, there was no significant difference in body weight in rapamycin treated as compared to placebo treated (23.5±1.3 g, n = 9 vs 23.0±1.3 g, n = 9, p = 0.12) normal mice. However, LV to body weight ratio was lower in rapamycin (3.0±0.1 mg/g; n = 9) than placebo (3.2±0.03 mg/g; n = 4; p = 0.01) treated normal mice.

**Figure 2:**
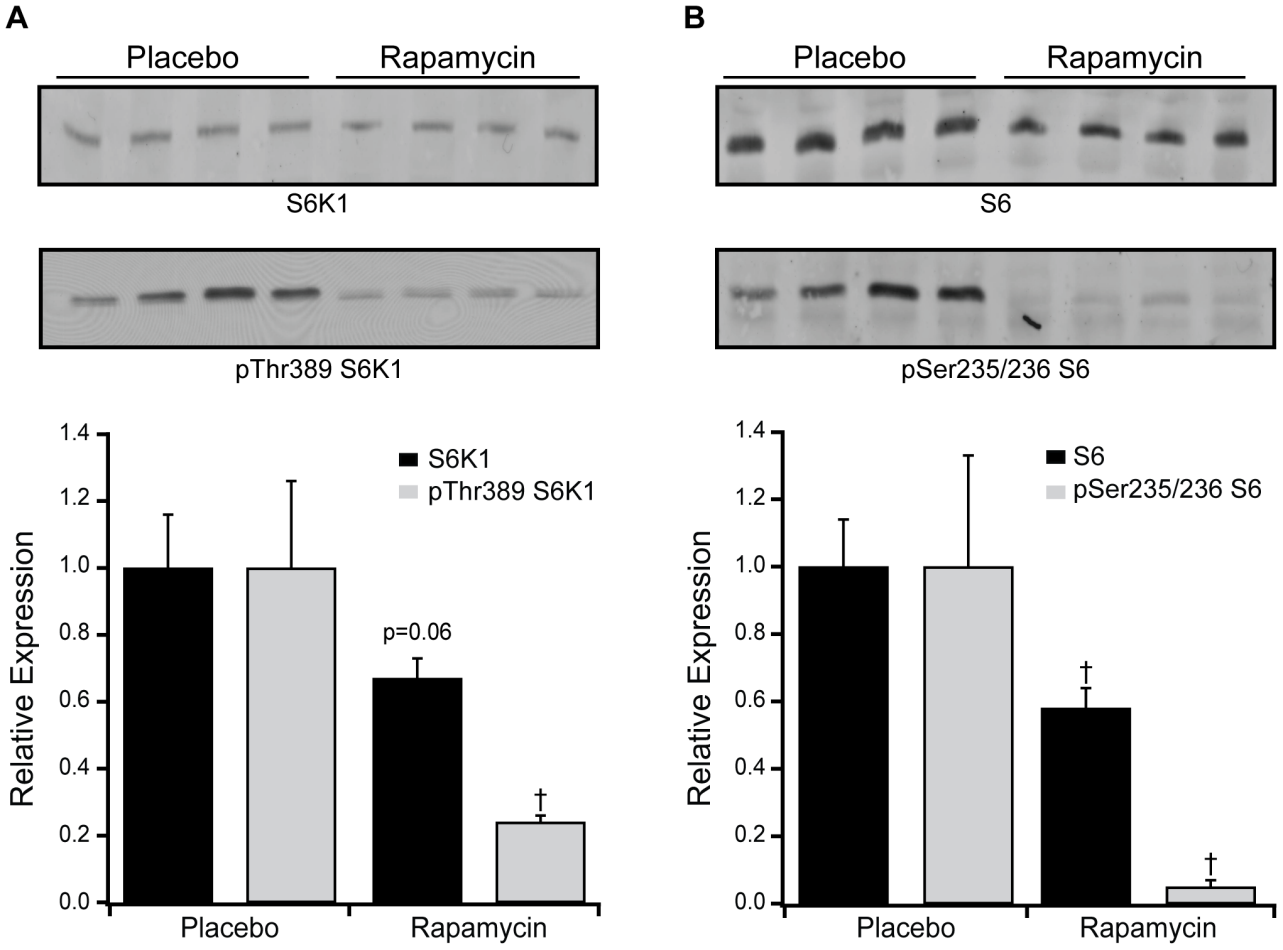
Effect of rapamycin on myocardial mTOR signaling in normal mice. Western blots and grouped data are shown for S6K1 and Thr389 phosphorylated S6K1 (A), or S6 and Ser235/236 phosphorylated S6 (B) in rapamycin (8 mg/kg/day PO, n = 4) versus placebo treated normal mice (n = 4). Rapamycin treatment showed a strong trend towards decreasing total S6K1 expression, whereas Thr389 phosphorylated S6K1 and total and Ser235/236 phosphorylated S6 were all significantly decreased. Data are mean ± SEM. †: *P*<0.05 vs. placebo.

Expression of total S6K1 trended (p = 0.06) lower while phosphorylation of S6K1 at Thr389 was lower in rapamycin versus placebo treated normal mice (Fig 2A). Expression of S6 and phosphorylation of S6 at Ser235/236 were both significantly lower in rapamycin versus placebo treated normal mice (Fig 2B).

**Figure 3:**
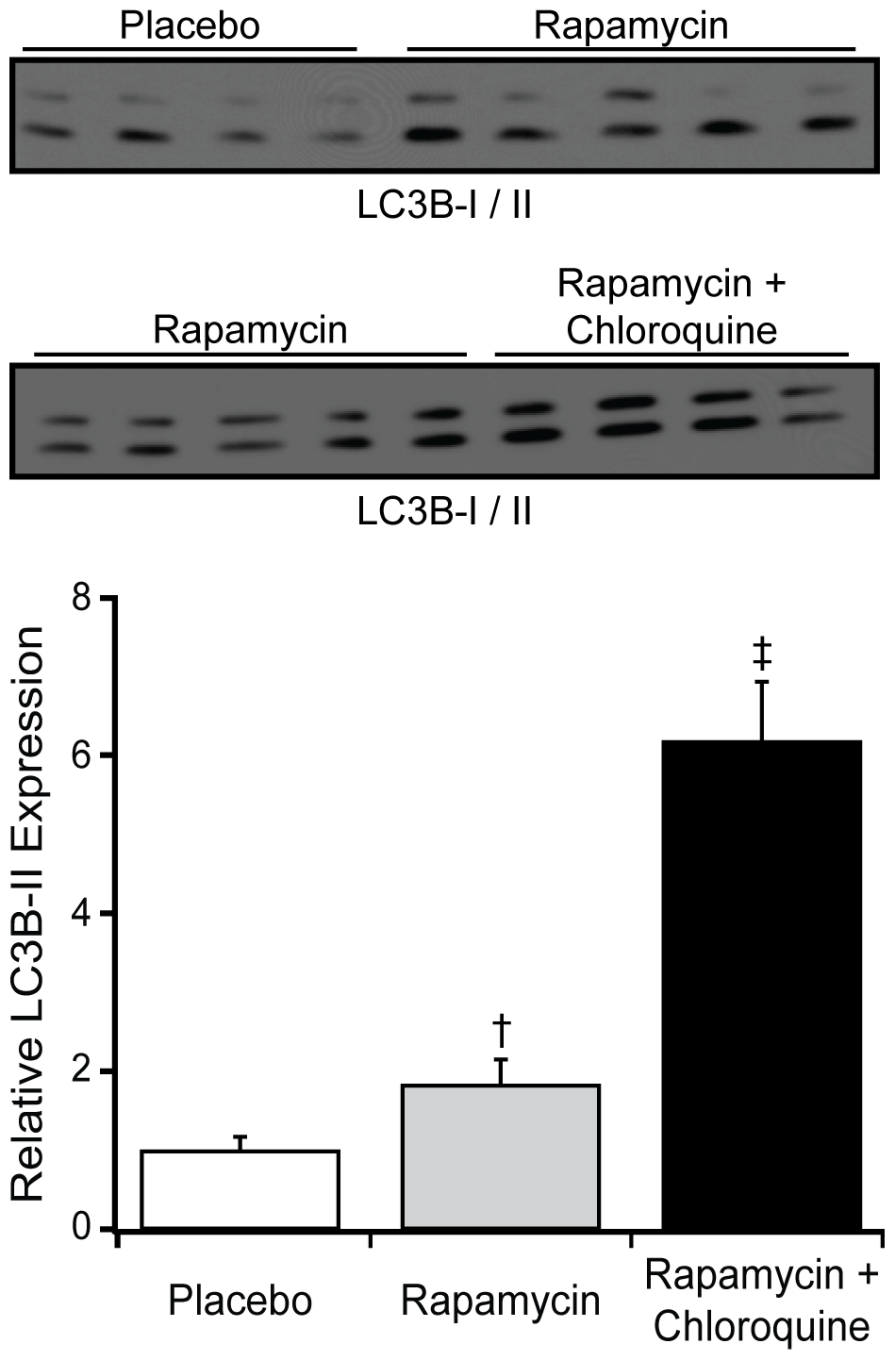
Effect of rapamycin on autophagy and autophagic flux in normal mice. Using an antibody that identified both LC3B-I (top band) and -II (bottom band), representative Western blots and grouped data are presented for myocardial LC3B-II levels in normal mice treated with placebo (n = 4), 8 mg/kg/day PO rapamycin (n = 5) or rapamycin plus chloroquine (10 mg/kg IP, n = 4) to assess autophagic flux. Data are mean ± SEM. †: *P*<0.05 vs placebo, ‡: *P*<0.05 vs. rapamycin.

As compared to placebo treated normal mice, rapamycin treated mice had higher LC3B-II expression with a further increase following the addition of chloroquine to rapamycin (Fig 3), consistent with rapamycin induced autophagy rather than the presence of lysosomal dysfunction [22].

### The HF model

**Figure 4:**
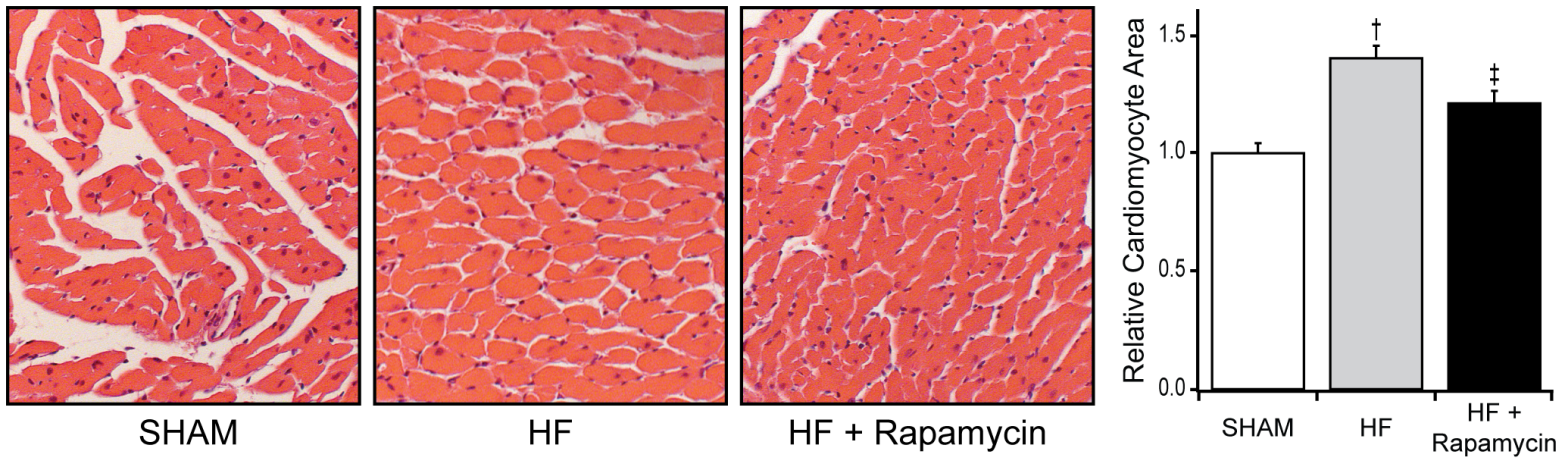
Effect of rapamycin on cardiomyocyte cross-sectional area in HF. Representative left ventricular myocardial sections and grouped data for cardiomyocyte area in SHAM operated (1±0.04, n = 5), placebo treated HF (1.40±0.05, n = 5) and 8 mg/kg/day PO rapamycin treated HF mice (1.21±0.05, n = 6). Data are mean ± SEM. †: *P*<0.05 vs SHAM, ‡: *P*<0.05 vs. placebo treated HF mice.

**Figure 5:**
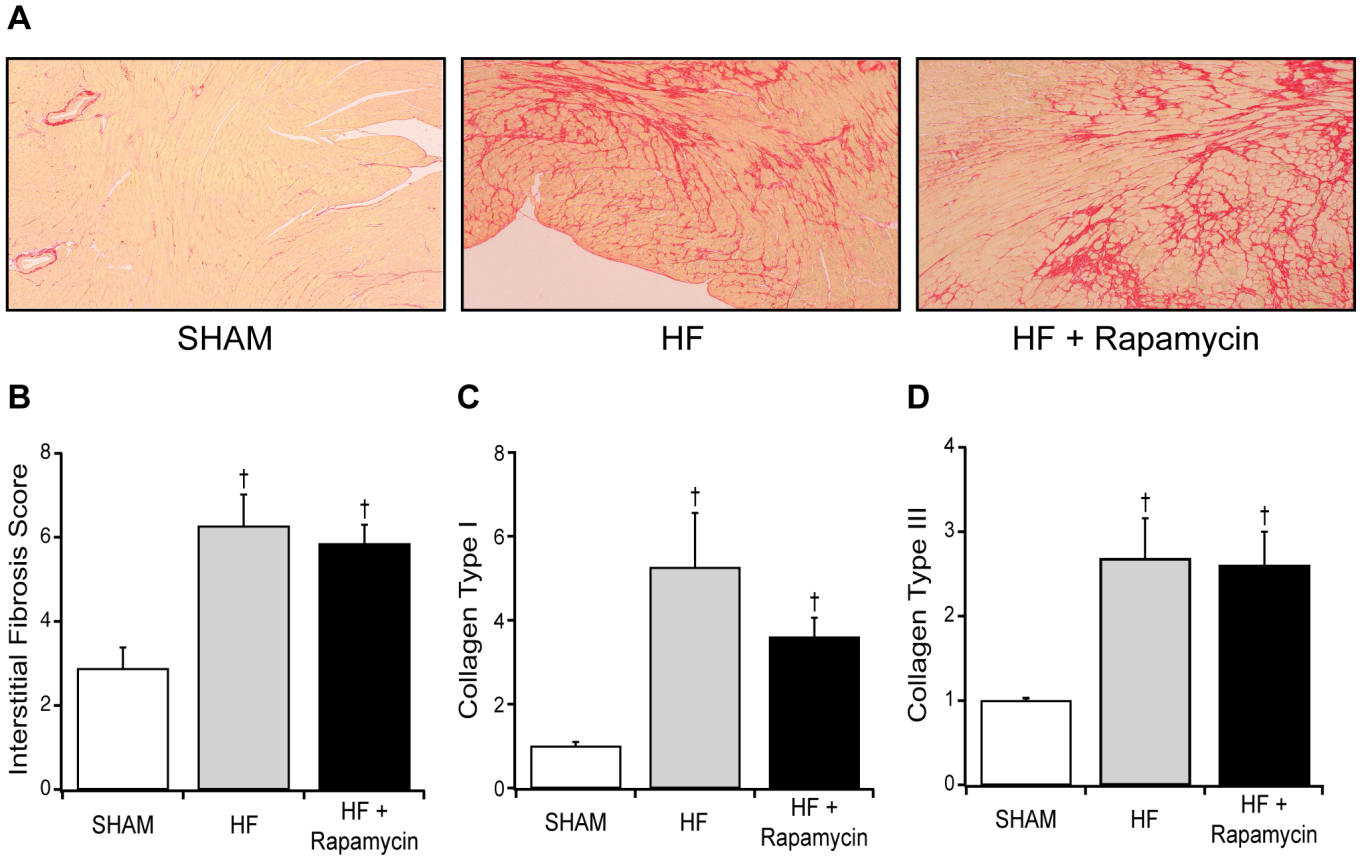
Effect of rapamycin on myocardial fibrosis in HF. Representative examples of picrosirius red stained left ventricular sections (A) and grouped data for interstitial fibrosis score (B) and relative collagen type I (C) and III (D) transcript levels in SHAM operated, placebo treated HF and 8 mg/kg/day PO rapamycin treated HF mice. Interstitial fibrosis scores were significantly higher in HF (n = 13) and HF + rapamycin mice (n = 11) when compared to SHAM (n = 8). For collagen type I and III transcripts, expression levels were significantly higher in HF (n = 5) and HF + rapamycin mice (n = 4) when compared to SHAM (n = 4). Data are mean ± SEM. †: *P*<0.05 vs SHAM.

As compared to SHAM, TAC mice with HF had decreased body weight, increased lung to body weight ratio consistent with pulmonary congestion, LV dilatation (increased LV end-diastolic dimension), and severe systolic dysfunction (lower EF) at 9 weeks post-TAC (Table 1). Left ventricular hypertrophy was present as evidenced by increased heart and LV to body weight ratios, upregulation of ANP gene expression (Table 1) and by increased cardiomyocyte cross-sectional area (Fig 4), although the cardiomyocyte cross-sectional area may be interpreted with caution as the hearts were not perfusion-fixed in end-diastole. Myocardial fibrosis was present as evidenced by higher fibrosis score and two- to five-fold increases in collagen I and III gene expression (Fig 5). Increased aortic flow velocity was present confirming significant TAC whereas the lack of increase in LV systolic pressure measured invasively reflects the severe systolic dysfunction (Table 1).

**Table 1:** Effect of rapamycin in TAC mice with heart failure (TAC-HF).

		Sham	TAC-HF	TAC-HF	TAC-HF
Treatment		NA	Placebo	Rapamycin	Rapamycin
Dose (PO)				8 mg/kg/day	4 mg/kg/day
Entering study (n)		14	17	17	9
Alive at end of study (n)		14	12	13	8
BW at study entry (g)		NA	22.5±0.4	24.6±0.5†	21.8±0.5*
Pre-treatment EF (%)		NA	48.8±2.3	52.9±2.4	50.0±3.9
Pathology					
	BW (g)	30.6±0.6	24.4±1.0†	28.1±0.8‡	25.7±0.6*
	HW/BW (mg/g)	4.7±0.2	12.3±0.9†	7.3±0.4‡	7.4±0.6‡
	LV/BW (mg/g)	3.2±0.1	8.1±0.5†	5.0±0.2‡	5.2±0.4‡
	Lung/BW (mg/g)	5.7±0.2	16.5±1.6†	9.0±0.9‡	8.2±1.2‡
Echocardiography					
	Aortic flow velocity (m/s)	1.1±0.4	2.8±0.9†	3.1±0.6	2.6±0.3
	EF (%)	80.3±1.3	30.4±4.9†	59.9±3.8‡	66.4±7.3‡
	LVEDD (mm)	3.7±0.3	4.9±0.6†	3.7±0.5‡	3.3±0.8‡
LVSP (mm•Hg)		108±4	125±5	152±9‡	143±12
ANP (AU/Sham)		1.0±0.3	29.5±5.4†	14.2±2.1‡	NA

BW: body weight; EF: ejection fraction; HW: heart weight; LV: left ventricular; LVEDD: Left ventricular end diastolic dimension; LVSP: LV systolic pressure; ANP: atrial natriuretic peptide; AU/Sham: arbitrary units indexed to Sham. †: *P*<0.05 vs Sham; ‡: *P*<0.05 vs TAC-HF placebo; *: *P*<0.05 TAC-HF + Rapamycin 8 mg/kg/day.

**Figure 6:**
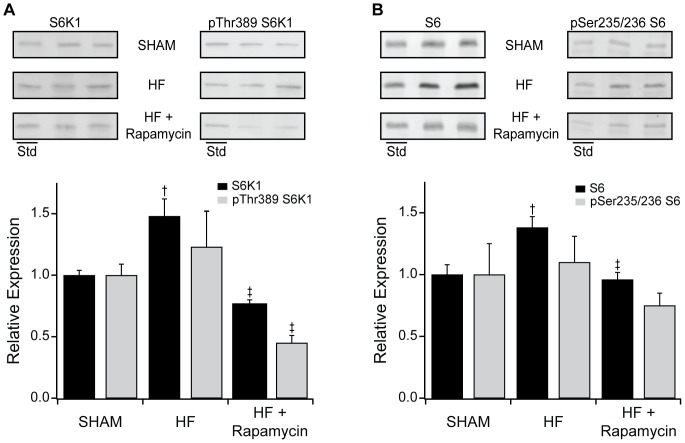
Effect of rapamycin on myocardial mTOR signaling in HF. Representative Western blots and grouped data for total or Thr389 phosphorylated S6K1 (A) and total or Ser235/236 phosphorylated S6 (B) in SHAM operated (n = 6), placebo treated HF (n = 6) and 8 mg/kg/day PO rapamycin treated HF mice (n = 6). Std: A standard LV homogenate sample for reliable comparison across gels. Data are mean ± SEM. †: *P*<0.05 vs SHAM, ‡: *P*<0.05 vs. placebo treated HF mice.

**Figure 7:**
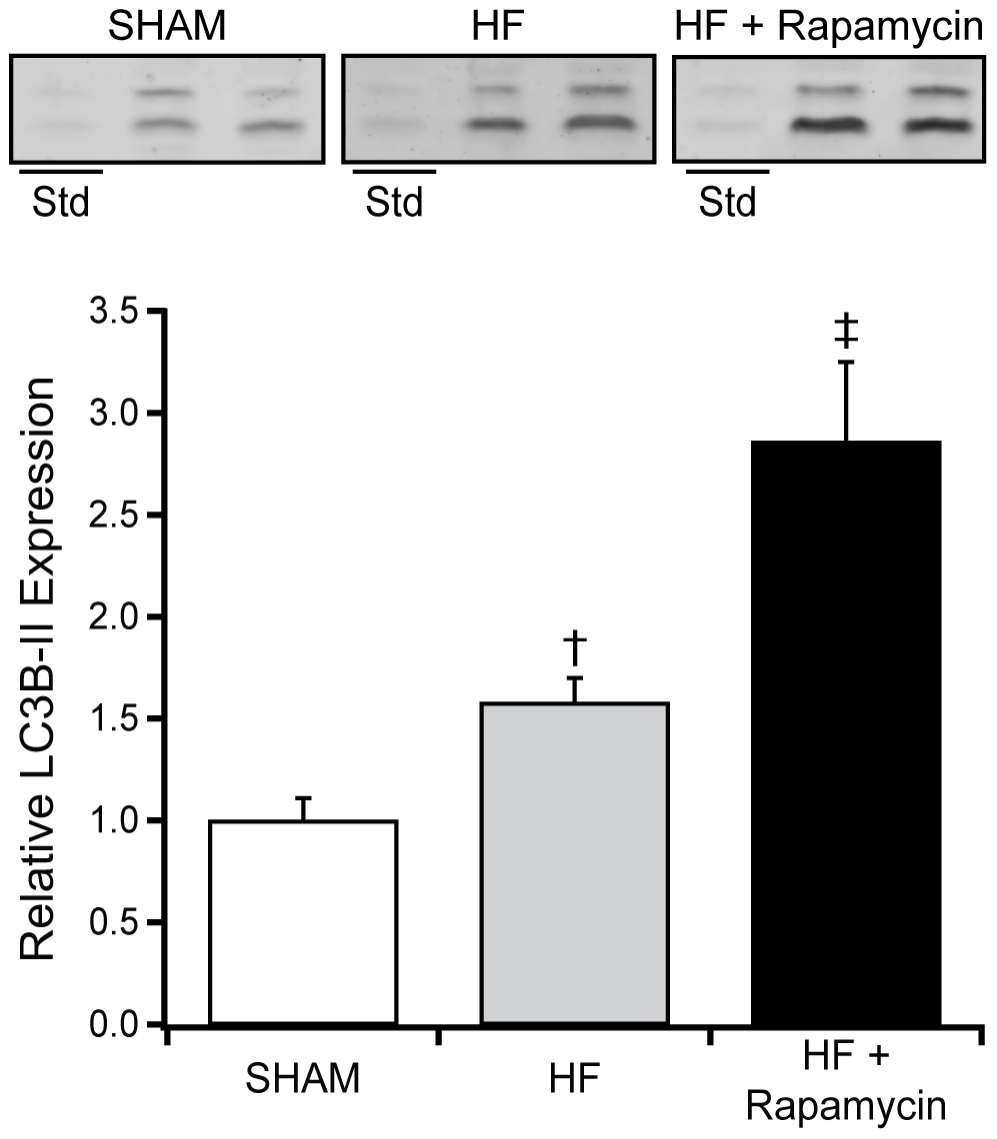
Effect of rapamycin on autophagy in HF. Representative Western blots and grouped data for LC3B-II expression (bottom band) in myocardium from SHAM operated (n = 6), placebo treated HF (n = 6) and 8 mg/kg/day PO rapamycin treated HF mice (n = 6). Std: A standard LV homogenate sample for reliable comparison across gels. Data are mean ± SEM. †: *P*<0.05 vs SHAM, ‡: *P*<0.05 vs. placebo treated HF mice.

As indexed to actin and compared to SHAM mice, abundance of total S6K1 and S6 but not Thr389 phosphorylated S6K1 or Ser235/236 phosphorylated S6 were increased in HF (Fig 6A and 6B). Compared to SHAM, HF mice had increased LC3B-II expression suggesting increased autophagy (Fig 7).

### Effect of target dose rapamycin in TAC mice with HF

Three weeks post-TAC, the pre-treatment EF was similar across all groups (Table 1). After 6 weeks of therapy, target dose rapamycin (8 mg/kg/day PO) produced immunosuppressive drug levels in HF (Fig. 1). As compared to placebo treated HF mice, target dose rapamycin treated HF mice had increased body weight, lower lung to body weight ratio, smaller LV end-diastolic dimension and higher EF (Table 1). Less hypertrophy was present as evidenced by lower heart and LV to body weight ratios, less upregulation of ANP gene transcription (Table 1) and by smaller cardiomyocyte cross-sectional area (Fig 4). However, myocardial fibrosis score and collagen type I and III mRNA transcript levels were not different from placebo treated HF mice (Fig 5).

Compared to placebo treated HF mice, abundance of both S6K1 and phosphorylation of S6K1 at Thr389 were lower in rapamycin treated HF mice (Fig 6B). Compared to placebo treated HF mice, abundance of S6 was reduced and phosphorylation of S6 at Ser235/236 tended to be reduced (p = 0.13) in rapamycin treated HF mice (Fig 6C). Compared to placebo treated HF mice, LC3B-II expression was increased in rapamycin treated HF mice (Fig 7).

### Effect of low dose rapamycin in HF mice

Treatment of HF mice with low dose rapamycin (4 mg/kg/day PO) resulted in trough rapamycin levels that were approximately 50% of those seen with the 8 mg/kg/day dose (Fig. 1). Compared to placebo treated HF mice, body weight and EF were higher whereas LV end-diastolic dimension and heart, LV and lung to body weight ratios were lower in the HF mice treated with low dose rapamycin (Table 1). There were no significant differences in these parameters between HF mice treated with the two different doses of rapamycin, save for body weight at TAC and at 9 weeks post-TAC, which were both lower in the low dose rapamycin group.

### Angiotensin receptor blockade without or with rapamycin in HF mice

As the effects of rapamycin were not dose related, the two dose groups were combined for comparisons to HF mice administered 30 mg/kg/day losartan PO or 30 mg/kg/day losartan plus 8 mg/kg/day PO rapamycin (Table 2). As compared to placebo treated HF mice, losartan treated mice had lower lung to body weight ratio, but there were no differences in body weight, heart or LV to body weight ratio or ejection fraction. Compared to rapamycin treated HF mice, losartan treated HF mice had higher heart to body weight and LV to body weight ratios.

**Table 2:** Effect of rapamycin and/or losartan in TAC mice with heart failure (TAC-HF).

		TAC-HF	TAC-HF	TAC-HF	TAC-HF
Treatment		Placebo	Rapamycin, both doses	Losartan	Losartan + Rapamycin
Rapamycin dose			4 or 8 mg/kg/day		8 mg/kg/day
Entering study (n)		17	26	13	11
Alive at end of study (n)		12	21	9	10
BW at study entry (g)		22.5±0.4	23.5±0.5	22.6±0.9	24.7±0.8
Pre-treatment EF (%)		48.8±2.3	51.7±2.0	45.0±4.8	50.9±2.3
Pathology					
	BW (g)	24.4±1.0	27.2±0.6†	24.7±1.1	28.1±1.0†
	HW/BW (mg/g)	12.3±0.9	7.3±0.3†	11.2±1.4‡	9.1±0.6†‡
	LV/BW (mg/g)	8.1±0.5	5.1±0.2†	7.7±0.9‡	6.2±0.3†‡
	Lung/BW (mg/g)	16.5±1.6	8.7±0.7†	11.1±1.8†	11.3±1.7†
Echocardiography					
	Aortic flow velocity (m/s)	2.8±0.9	2.9±0.7	2.5±0.8	3.5±0.9
	EF (%)	30.4±4.9	62.4±3.6†	49.1±9.8	41.5±5.6‡
	LVEDD (mm)	4.9±0.6	3.6±0.6†	4.3±0.9	4.5±0.6‡
LVSP (mm•Hg)		125±5	149±7†	131±8	137±7

BW: body weight; EF: ejection fraction; HW: heart weight; LV: left ventricular; LVEDD: Left ventricular end diastolic dimension; LVSP: LV systolic pressure. †: *P*<0.05 vs TAC-HF + placebo and ‡: *P*<0.05 vs TAC-HF + Rapamycin.

As compared to placebo treated HF mice, mice treated with rapamycin + losartan had lower heart, LV and lung to body weight ratios, although decreases in LV end-diastolic dimension and increases in ejection fraction with combination therapy did not achieve statistical significance. Compared to rapamycin treated HF mice, rapamycin + losartan treated mice had higher heart to body weight and LV to body weight ratios, increased LV end-diastolic dimension and lower ejection fraction.

Protein analyses demonstrated that total and Thr389 phosphorylated S6K1 expression as well as total and Ser235/236 phosphorylated S6 were not changed further by the addition of losartan to rapamycin (data not shown).

Survival after 6 weeks of therapy in rapamycin treated mice (pooled 4 and 8 mg/kg/day dose; Table 2) was numerically higher (81%) than in placebo treated mice (71%) or losartan treated mice (69%) but this difference was not statistically significant.

### Rapamycin and/or angiotensin receptor blockade in TAC-COMP

As compared to SHAM, at 9 weeks post TAC, placebo treated TAC-COMP mice had a significant reduction in body weight accompanied by increased heart and LV to body weight ratios, similar lung to body weight ratio, similar LV end-diastolic dimensions and a mild but significant reduction in ejection fraction consistent with a compensated state (Table 3). Although both rapamycin doses similarly decreased myocardial remodeling and improved ejection fraction in the TAC-COMP mice (Table 3), losartan alone did not have favorable effects on hypertrophy or systolic function, nor did the addition of losartan augment the beneficial effects of rapamycin (Table 4). Further, survival after 6 weeks of therapy was not different between any treatment group (Table 4)

**Table 3:** Effect of rapamycin in TAC mice with compensated hypertrophy (TAC-COMP).

		Sham	TAC-COMP	TAC-COMP	TAC-COMP
Treatment		NA	Placebo	Rapamycin	Rapamycin
Dose (PO)				8 mg/kg/day	4 mg/kg/day
Entering study (n)		14	13	9	9
Alive at end of study (n)		14	13	9	8
BW at study entry (g)		NA	22.4±0.4	24.3±0.3‡	22.6±0.4*
Pre-treatment EF (%)		NA	78.9±1.9	76.4±1.1	82.3±2.9
Pathology					
	BW (g)	30.6±0.6	27.4±0.5†	30.2±0.6‡	26.9±0.7*
	HW/BW (mg/g)	4.7±0.2	7.4±0.5†	5.6±0.3‡	6.4±0.2‡
	LV/BW (mg/g)	3.2±0.1	5.3±0.4†	3.9±0.3‡	4.6±0.2
	Lung/BW (mg/g)	5.7±0.2	6.3±0.4	6.4±0.4	6.1±0.3
Echocardiography					
	Aortic flow velocity (m/s)	1.0±0.1	2.4±0.2†	2.6±0.2	3.0±0.1‡
	EF (%)	80.3±1.3	64.2±4.4†	72.7±2.1‡	81.4±3.0‡
	LVEDD (mm)	3.7±0.1	3.5±0.2	3.9±0.1	2.8±0.2‡*
LVSP (mm•Hg)		108±4	176±19†	151±11	160±11

BW: body weight; EF: ejection fraction; HW: heart weight; LV: left ventricular; LVEDD: Left ventricular end diastolic dimension; LVSP: LV systolic pressure. †: *P*<0.05 vs Sham; ‡: *P*<0.05 vs TAC-COMP + placebo; *: *P*<0.05 vs TAC-COMP + Rapamycin at 8 mg/kg/day.

**Table 4:** Effect of rapamycin and/or losartan in TAC mice with compensated hypertrophy (TAC-COMP).

		TAC-COMP	TAC-COMP	TAC-COMP	TAC-COMP
Treatment		Placebo	Rapamycin, both doses	Losartan	Rapamycin + Losartan
Rapamycin dose			4 or 8 mg/kg/day		8 mg/kg/day
Entering study (n)		13	18	14	8
Alive at end of study (n)		13	17	12	7
BW at study entry (g)		22.4±0.4	23.5±0.3†	23.8±0.7	22.4±0.4
Pre-treatment EF (%)		78.9±1.9	79.3±1.7	75.8±1.5	75.9±2.2
Pathology					
	BW (g)	27.4±0.5	28.7±0.6	27.8±0.6	25.7±0.5
	HW/BW (mg/g)	7.4±0.5	6.0±0.2†	6.7±0.2	6.0±0.1†
	LV/BW (mg/g)	5.3±0.4	4.2±0.2†	4.7±0.2	4.3±0.1†
	Lung/BW (mg/g)	6.3±0.4	6.3±0.2	6.9±0.7	6.7±0.2
Echocardiography					
	Aortic flow velocity (m/s)	2.4±0.2	2.8±0.1	2.9±0.2†	2.9±0.3
	EF (%)	64.2±4.4	76.8±2.1†	71.7±5.5	72.2±2.7
	LVEDD (mm)	3.5±0.2	3.4±0.2	3.1±0.1	3.0±0.2
LVSP (mm•Hg)		176±19	155±7	149±9	144±9

BW: body weight; EF: ejection fraction; HW: heart weight; LV: left ventricular; LVEDD: Left ventricular end diastolic dimension; LVSP: LV systolic pressure. †: *P*<0.05 vs TAC-COMP + placebo.

## Discussion

In this study, rapamycin administered to mice with established HF attenuated progression of systolic dysfunction and hypertrophy but not fibrosis in association with expected effects on mTORC1 down-stream effectors (S6K1 and S6) and enhanced autophagy as assessed by LC3B-II levels. The beneficial effects of rapamycin were noted at *and below* serum levels that are targeted for primary immunosuppression. Treatment with rapamycin was superior to but not incrementally improved by ARB. Findings were similar in mice with compensated LV hypertrophy. As the anti-remodeling effects of rapamycin are evident at low dose and are superior to standard HF therapy in the presence of continued cardiac stress, these data lend support for investigation of low dose mTOR inhibition in advanced HF.

### Effect of mTOR inhibition with rapamycin on pathologic remodeling in established HF

In a previous study, a seven day course of rapamycin treatment started one week after TAC surgery attenuated adverse remodeling and dysfunction in mice with decompensated hypertrophy [18]. Our study supports these findings while extending them in several important ways. In the current study, rapamycin treatment was started three weeks after TAC while treatment duration was extended to six weeks. Despite the more advanced stage of disease, six weeks of rapamycin treatment attenuated progressive ventricular dysfunction, remodeling, and pulmonary congestion. These findings demonstrate that more established HF is not resistant to mTOR inhibition and that longer term therapy is not associated with adverse cardiac effects.

This study also characterized the effects of rapamycin treatment on fibrosis in established HF, but no beneficial effect was seen regardless of rapamycin dose level (Fig. 5). In a murine TAC model unassociated with HF [16] and in a transgenic model of prolonged AKT overexpression [8], rapamycin therapy was observed to attenuate fibrosis. Although hypertrophic signaling and inflammation are linked to myocardial fibrosis, rapamycin did not reduce fibrosis in progressive HF despite its ability to attenuate some of these signaling inputs [29], [30]. It is possible that in this model of TAC-HF, the well-established fibrosis prior to drug administration would necessitate longer treatment time to resolve, similar to that observed after relief of pressure overload in humans [31]. Alternatively, rapamycin may not effectively modulate intracellular signaling pathways more strongly associated with pro-fibrotic conditions, such as increased transforming growth factor-β, connective tissue growth factor or Rho-mediated signaling [29]. Finally, the assessment of fibrosis utilized here did not distinguish between effects on reactive versus reparative fibrosis.

Despite the persistent myocardial fibrosis, the observed reduction in end diastolic chamber dimension and improved ejection fraction despite persistent pressure overload clearly indicate that rapamycin treatment attenuated further impairment in contractility. Pharmacologic or genetic antagonism of several of the intracellular pathways activated in pathological hypertrophy or forced over-expression of anti-hypertrophic factors have been associated with coincident amelioration of hypertrophy and systolic dysfunction in rodent models of pressure overload (reviewed in [32]). The mechanisms responsible for these seemingly related phenomena are unclear but promotion of a balance between angiogenesis and hypertrophy [8] and reduction in hypertrophy associated pro-apoptotic factors [32] have been suggested to mediate the improvement in contractility associated with reduction in hypertrophy. Others have suggested that rapamycin administration ameliorates adverse changes in gene expression in heart failure, specifically preserving α-myosin heavy chain and SERCA 2a gene expression, both of which are associated with improved myocardial function [18].

Our study is also the first to explore the dose response of rapamycin in experimental HF, primarily because clinical translation would benefit from reduced dose administration of this known immunosuppressant. Prior studies in experimental pressure overload have used 2 mg/kg/day administered by IP injection [18], [33], a dose that results in serum rapamycin levels within the target range used for primary immunosuppression in humans (Fig. 1; [11]). In cardiac transplantation patients converted from calcineurin inhibitors to rapamycin as their primary immunosuppressant, these serum levels provided for regression of LV hypertrophy [34]. Because rapamycin is poorly absorbed in rodents [35], a dose of 8 mg/kg/day PO was required to produce serum levels similar to those achieved with 2 mg/kg/day IP (Fig. 1). In our TAC-HF model, the anti-remodeling and cardiac contractility improvements over placebo were confirmed at 8 mg/kg/day PO rapamycin and, more importantly, were retained at the 4 mg/kg/day PO dose despite producing serum concentrations that were approximately half those targeted for immunosuppression (Table 1; Fig. 1). Further, the benefit of rapamycin treatment was evident even with the sustained load in the TAC-HF model, suggesting that potential load effects involved in switching cardiac transplantation patients from calcineurin inhibitors to rapamycin were not the major drivers for the regression of LV hypertrophy.

### Effect of rapamycin on mTOR signaling in established HF

In this murine HF model, the downstream mTOR signaling proteins S6K1 and S6 were both increased in TAC-HF, although the abundance of Thr389 phosphorylated S6K1 and Ser235/236 phosphorylated S6 were not increased in HF (Fig. 6). The reduction in the abundance of total and phosphorylated S6K1 in normal mice with rapamycin confirms mTOR inhibition with treatment (Fig. 2) and the significant decline in phosphorylated S6K1 in TAC-HF mice treated with rapamycin suggests that mTOR signaling through this pathway remains active in HF and is susceptible to regulation [36]. In normal mice, rapamycin administration was associated with marked decreases in total S6 and phosphorylated S6 abundance, as expected due to reduced S6K1 activation. In rapamycin treated TAC-HF mice, total S6 expression was lower along with a trend towards decreased S6 phosphorylation. Reduction in S6 is only one of the S6K1 regulated pathways affecting protein synthesis after mTORC1 activation and preferential downstream targets of S6K1 may be altered during TAC-HF induced myocardial remodeling. However, at this point we do not have data resolving the relative contributions of S6K1 versus autophagy in the observed remodeling, nor can we exclude the effect of mTOR inhibition on cardiomyocyte morphology, the contractile apparatus or the composition of the extracellular matrix.

In addition to its effects on protein synthesis, mTOR activation suppresses autophagy whereas its inhibition increases autophagy [37]. This study, to our knowledge, is the first to examine the effect of rapamycin therapy on autophagy markers in experimental pressure overload induced HF. Rapamycin treatment with and without accompanying chloroquine administration in normal mice produced the expected effect on LC3B-II expression and confirmed that these were likely related to true pro-autophagic events (Fig. 3). Higher LC3B-II levels were indicative of increased autophagy in TAC-HF mice, and these levels were further increased by rapamycin treatment (Fig. 7). Increases in autophagy with rapamycin may contribute to its effects on cardiac remodeling. Although autophagy may exert both protective and detrimental effects in cardiovascular disease, in the proper context, enhancing autophagy may provide ATP production, clearance of oxidized proteins and damaged organelles including dysfunctional mitochondria and thus enhance myocardial metabolism and performance [38], [39]. Indeed, autophagy appears to be a critical factor for optimal cardiac remodeling in response to stress, as cardiac specific deletion of autophagy related protein-5 leads to rapid progression to decompensation in TAC mice [40].

It deserves noting that studies with pharmacologic mTOR inhibition are distinct from studies of cardiac-specific ablation of mTOR or raptor, a key component of the mTORC1 complex which mediates effects on protein synthesis and autophagy via S6K1 and 4E binding protein. In both mTOR and raptor deletion models, targeted ablation of mTOR signaling in the heart prior to pressure overload has been associated with cardiac dilatation, systolic dysfunction and early mortality [33], [41]. While these findings may indicate that marked mTOR inhibition prevents the ability to mount an initial compensatory hypertrophic response to pressure overload, mTOR inhibition with rapamycin started at the time of or before cardiac stress [12], [21], [42] was not associated with such adverse effects. It is unclear whether the *mode* (genetic vs pharmacologic), the *degree* (complete vs partial) or the *site* (systemic vs cardiomyocyte-restricted) of mTOR inhibition mediate the disparate effects on cardiac remodeling in the genetic ablation versus pharmacologic inhibition studies. In zebrafish cardiomyopathy models, haploinsufficiency of TOR or rapamycin administration both improved cardiac function, prevented pathological remodeling events and reduced mortality [43]. Beneficial effects of partial mTOR inhibition either with rapamycin or through haploinsufficiency may suggest that some degree of mTOR activity is sufficient for compensatory hypertrophy, but that partial inhibition attenuates excessive mTOR activity that may drive deleterious remodeling in HF. Further studies are needed to understand the differences between genetic and pharmacologic mTOR inhibition, the site of mTOR inhibition and its timing relative to cardiac stress.

### Angiotensin II receptor blockade with or without mTOR inhibition in TAC-HF mice

*In vitro* studies in cultured ventricular myocytes show that angiotensin II, along with α-adrenoceptor and β-adrenoceptor agonism induce protein synthesis and hypertrophy via activation of S6K, and can be antagonized by mTOR inhibition with rapamycin [13], [14], [44]. The effect of ARB on remodeling in rodent pressure overload models has been mixed with some studies showing that ARB produced modest reversal of adverse remodeling and LV hypertrophy [45]–[47] while other studies have not found any benefit [48]–[51]. Further, ablation of the angiotensin II receptor did not attenuate the response to TAC in mice [52]. In humans, ARB clearly opposes hypertrophic remodeling in association with blood pressure reduction although it is commonly accepted that the beneficial effects of ARB are not completely explained by reduction in blood pressure [53]. Although the TAC mouse model may be considered unique in that mechanical stress persists despite ARB therapy, this situation is not unlike that in patients wherein wall stress is markedly increased in HF even when blood pressure is controlled, owing to the marked cardiac dilatation and wall thinning, which potently contribute to wall stress [54]. The contribution of mechanical stress versus direct myocardial effects of angiotensin II to the hypertrophic response is difficult to distinguish and may change according to the stage of HF. Thus, the lack of benefit observed with ARB here may suggest that in this particular model, direct effects of angiotensin receptor stimulation contribute less to hypertrophic remodeling than continued hemodynamic stress.

We noted some attenuation of rapamycin's beneficial effects on EF, HW:BW and LV:BW ratios by losartan in TAC-HF. However, losartan did not blunt the reverse remodeling effects of rapamycin in mice with compensated hypertrophy. This may reflect chance differences in the severity of TAC in the losartan + rapamycin treated HF mice. While we endeavored to ensure similar baseline characteristics by our study entry criteria (3 week ejection fraction <65%), the losartan + rapamycin group may have had more severe TAC as noted by a trend towards higher aortic flow velocities at 6 weeks (without higher ejection fraction). Further, we cannot rule out the possibility that cross-talk between signaling pathways was altered in the presence of ARB, potentially antagonizing rapamycin's effect by reactivating Akt signaling [55], [56]. Nonetheless, the absence of strong antagonism between ARB and rapamycin treatment and the more potent effects of rapamycin in this model and animal studies with rapamycin in other forms of experimental HF (myocardial infarction, prolonged Akt overexpression) support the translational effort. However, studies in higher mammalian species along with correlations between myocardial remodeling effects, drug levels and accompanying markers of immunosuppression, would strengthen the rationale for human translation.

## Conclusions

mTOR inhibition with rapamycin, using doses that produce drug levels at or below those targeted when rapamycin is used for primary immunosuppression, attenuated adverse cardiac remodeling in a mouse model of established HF. Anti-remodeling effects of rapamycin were superior to and not enhanced by therapy with a neurohumoral antagonist. These data lend support for the potential clinical investigation of low dose mTOR inhibition in advanced HF.
